# COVID-19 Effect on Access to Maternal Health Services in Kenya

**DOI:** 10.3389/fgwh.2020.599267

**Published:** 2020-11-26

**Authors:** Jackline Oluoch-Aridi, Tecla Chelagat, Mary M. Nyikuri, Joseph Onyango, Danice Guzman, Cindy Makanga, Laura Miller-Graff, Robert Dowd

**Affiliations:** ^1^The Ford Family Program in Human Development Studies and Solidarity, Kellogg Institute for International Studies, University of Notre Dame, Notre Dame, IN, United States; ^2^Strathmore Business School, Institute of Healthcare Management, Strathmore University, Nairobi, Kenya; ^3^Department of Psychology, The Kroc Institute of Peace Studies, University of Notre Dame, Notre Dame, IN, United States

**Keywords:** COVID-19, informal settlements, Kenya, women, maternity services, access

## Abstract

**Introduction:** Maternal mortality continues to be one of the biggest challenges of the health system in Kenya. Informal settlements in Kenya have been known to have higher rates of maternal mortality and also receive maternity services of varied quality. Data assessing progress on key maternal health indicators within informal settlements are also often scarce. The COVID-19 pandemic hit Kenya in March this year and so far, the impact of the pandemic on access to maternal health has not been established. This study aims to add to the body of knowledge by investigating the effects of the COVID-19 pandemic and mitigation strategies on access to health care services in informal settlements.

**Methods:** Qualitative methods using in-depth interviews were used to assess women's experiences of maternity care during the COVID-19 era and the impact of proposed mitigation strategies such as the lockdown and the curfew. Other aspects of the maternity experience such as women's knowledge of COVID-19, their perceived risk of infection, access to health facilities, perceived quality of care were assessed. Challenges that women facing as a result of the lockdown and curfew with respect to maternal health access and quality were also assessed.

**Results:** Our findings illustrate that there was a high awareness of the symptoms and preventative measures for COVID-19 amongst women in informal settlements. Our findings also show that women's perception of risk to themselves was high, whereas risk to family and friends, and in their neighborhood was perceived as low. Less than half of women reported reduced access due to fear of contracting Coronavirus, Deprioritization of health services, economic constraints, and psychosocial effects were reported due to the imposed lockdown and curfew. Most respondents perceived improvements in quality of care due to short-waiting times, hygiene measures, and responsive health personnel. However, this was only reported for the outpatient services and not in-patient services.

**Conclusion:** The most important recommendation was for the Government to provide food followed by financial support and other basic amenities. This has implications for the Government's mitigation measures that are focused on public health measures and lack social safety-net approaches for the most vulnerable communities.

## Introduction

The Novel Coronavirus disease, commonly referred as COVID-19, was declared a public health emergency of international concern on 30th January 2020 and declared a global pandemic on the 11th March 2020 (1). Previous research has indicated that pandemics, such as Ebola in West Africa, can devastate the provision of maternal health services in low-resilience health systems (2–4). A study modeling the coverage of essential maternal and child health interventions estimated a 8.3–38.6% increase in maternal deaths per month across 118 low- and middle-income countries (LMICs) during the COVID-19 pandemic (5). The COVID-19 pandemic reached Kenya on March 15, 2020. Currently (as per 25th August 2020) the number of cases in Kenya is estimated at 32,577 infections, with 18,895 recoveries and 554 deaths (6). Nairobi City in Kenya is estimated to have the highest the number of COVID infections in the country with at 342 as per July 2020 with ~30% of new infections in peri-urban settings (7). It is estimated that about 70% of Nairobi's more than 4 million residents reside in urban slums (8–10).

As a preventive response, Kenya imposed strict curfews and lockdown rules to prevent the spread of COVID-19. The Kenyan Government banned international flights, closed schools, and banned large social gatherings; mass prayer gatherings, large weddings and funerals, in order to prevent the accelerated transmission of the virus (11). In addition to this, the Government issued a 30-day lock down as a mitigation measure to COVID-19 transmission. This was accompanied by a curfew that was initially restricted movement between 7.00 p.m. and 4.00 a.m. but was subsequently extended to 9.00 p.m. to 5.00 a.m. These government directives pose a huge dilemma, as they have disrupted access to health services by mothers (12). Despite the fact that expectant mothers have been allowed to leave their houses and go to health facilities to access delivery care during emergencies. They have however had challenges with transportation to health facilities during the curfew. These restrictions may disproportionately affect those living in informal settlements within large cities. Recent evidence has shown that women face access challenges that are both structural, such as transportation to health facilities, and social, such as fear of health care workers (13). These settings also have already existing challenges with regard to the quality of maternal health care services and women report receiving maternal health services of varied quality (13).

Studies that have focused in informal settlements have assessed residents' perceptions of government directives to contain the pandemic and concluded that the Government measures to mitigate COVID-19 need to have communication channels that are targeted at reaching less-educated households (14). Other studies have shown that although COVID-19 mitigation strategies are needed, they are known to have an indirect negative impact on women's well-being, for example many hospitals have put restrictions on visits by partners and relatives during admission at the hospitals for delivery (15). This is despite the fact that women are normally denied companionship during delivery in most hospitals due to privacy concerns (16). The COVID period presents new challenges for women with the lockdowns and curfews instilling fear and necessitating the need for birth companionship. Lockdowns have also result in varied economic consequences such as job loss, food and housing insecurity further aggravating health outcomes (17, 18). It is possible that within informal settlements the impact of the job losses might indirectly affect women's ability to access health care due to economic hardship, subsequently reducing access to health facilities.

A review of the existing literature demonstrates an information gap on the impact of pandemic on maternal well-being especially in a resource-scarce setting where marginalized women often receive poor quality of care (19, 20). A population survey in 60 LMICs suggest a declining trend on utilization of maternal and child health (MCH) services such as delivery, antenatal care (ANC) attendance and child immunization (21). Studies have established that health workers in other sub-Saharan African settings are not well-prepared to provide treatment for COVID patients and insufficiently prepared to meet the demands of the women (22). These limitations may have serious implications for women's health. For example, a recent study modeling the coverage of essential maternal and child health interventions estimated a 8.3–38.6% increase in maternal deaths per month across 118 low- and middle-income countries (LMICs) during the COVID-19 pandemic (5).

This study aimed to assess the extent of the impact of the imposed lockdowns and curfew on access to maternal health services for women living in informal settlements. We also assessed women's knowledge of the signs and symptoms of COVID-19, women's perceived risk of infection to further understand how the virus affects women and their health during the pandemic. These findings provide a critical information for frontline health workers and policy makers who are seeking to quickly develop pandemic responsive programs and strategies that are relevant, person-centered and context friendly. By incorporating women's voices, Ministries of Health and other non-state health care providers can be better able conduct targeted care and minimize the negative consequences of COVID-19.

## Methods

### Study Setting

This qualitative study focused on women's experiences with person-centered maternity care amongst women living in the informal settlements in the Embakasi area in Nairobi City, Kenya. The study area has an estimated population of almost one million people, in mostly low-income housing and informal settlements. Residents in Embakasi experience widespread poverty and high unemployment and belong to the lowest wealth quintile in Kenya. The health system consists of both primary public health centers and several private health facilities and mission health facilities. The main referral health facility is a secondary maternity hospital.

### Data Collection

#### Study Design, Recruitment, and Participants

Participants were drawn from an ongoing longitudinal study focused on assesses quality of maternal care services in the Embakasi region of Nairobi, capital of Kenya. For the current study, a subsample of women was selected to completed telephonic interviews (71), which were conducted by four researchers in the months May-June 2020 with women who had received services for childbirth in the past 6 weeks from public, private and missionary hospitals in this region.

The first author is a trained public health specialist and the research assistants were trained on qualitative research methods. The facilities were purposively selected to represent both health centers and secondary maternities. They also represented three types of health facilities present in Kenya: public, private, and mission health facilities. The health facilities in the study were chosen in collaboration with the first author.

Women were recruited during child welfare clinics. The inclusion criteria were women who were aged between 18 and 49 and had delivered their babies within the identified facilities in the past 6 weeks. Verbal informed consent was obtained from all the women after providing information about the study and the potential benefits and risks of their involvement in the study via a phone call. The interviews were conducted by phone to mitigate the risk to participants due to COVID-19. During the phone interviews women were asked whether they had been to the health facility during the COVID-19 pandemic and to share their experiences during this time. A semi-structured interview guide was used (See Appendix 1). Interviews were conducted in Kiswahili, a language commonly spoken by women in this setting. The discussions were recorded and transcribed verbatim in Kiswahili. The transcripts were then, translated into English by the sixth author (CM) who is a native speaker of Kiswahili. The transcripts were back translated from Kiswahili to English by the first author also a native speaker of Kiswahili to ensure that the meaning was maintained. A total of 82 women who met the criteria were approached via phone calls. Nine of them were in an area with very poor mobile phone connectivity. Two women were unavailable. Therefore only 71 interviews were conducted. Ethical review approval from Strathmore University IRB, University of Notre Dame IRB and permission to conduct the research from The National Commission on Science Technology and Innovation (NACOSTI).

#### Data Analysis

We read all the transcripts to gain familiarization with the data. We iteratively coded line-by-line across the entire data set. We then analyzed the data applying emerging codes. We then compared these codes to those in the coding framework that we established a priori from the interview guide. We followed Braun and Clark's (23) thematic analysis to analyze the data. We grouped codes into categories, reviewed the themes for patterns, defined the themes. Four coders compared the themes and discussed the themes. We reviewed transcripts and analyzed the data until we reached data saturation and we could not identify any new themes. An Additional Appendix 2 to show how open codes were used to generate categories and themes.

## Results

The characteristics of the women respondents are contained in Table 1 below.

**Table 1:** Sociodemographic characteristics of the participants.

**Participant characteristics**	**Percentage *N =* 71**
Age (Mean)	28 (5.3)
**Parity**	
Primiparous	16 (23%)
Multiparous	55 (77%)
**Marital Status**	
Married	61 (86%)
Single	10 (14%)
**Education**	
Primary	18 (25%)
Secondary	43 (61%)
College	10 (14%)
**Occupation**	
Employed	10 (14%)
Unemployed	61 (86%)
**Type of health facility**	
Mission health facility	29 (41%)
Public health facility	25 (53%)
Private health facility	17 (24%)
**Delivery history**	
All deliveries in a health facility	61 (86%)
At least one delivery outside health facility	10 (14%)

The mean age of the women was 28 years. Seventy seven percentage of the women were multiparous with a majority (88%) of the women were married. A vast majority of the women were unemployed (86%). The rest of the characteristics are contained in Table 1. We identified four main themes in the data: (1) Awareness and risk perception on COVID-19. In this theme we discuss women's ability to identify at least three symptoms of COVID-19, key prevention measures as well as their perceived risk of infection (2) perceived quality of health services. This theme describes our findings regarding women's perceived changes in the quality of maternal health services during the COVID-19 pandemic. (3) Economic challenges. This theme identifies women's several accounts of economic struggles where they describe their experiences with loss of income generation and this resulting in their in ability to afford transportation to the health facility and (4) mitigation strategies. This theme identifies strategies that women used to try and mitigate the impacts of the COVID-19 pandemic that would allow them to increase access to health facilities.

### Awareness and Risk Perception on COVID-19

Most of the respondents with about 60% had a high awareness of the key symptoms and preventative measures employed to reduce the spread of the COVID-19. They were able to mention key symptoms such as high body temperature, persistent cough, and fever. Most women were aware of preventative measures including frequent washing of hands, sanitizing, wearing face masks and maintaining social distance.

“*...The symptoms include: fever, severe coughing and also an increase in the body temperature. I know it is a dangerous disease whose preventive measures include: frequent washing of hands, sanitizing, wearing face masks and maintaining social distance of one meter away when interacting with other people…” (Respondent #44)*

### Perceived Risk of Contracting COVID-19

Respondents were asked about their risk of contracting COVID-19 to self, family and friends and their neighborhood. A majority of the women—~56% of those women interviewed—perceived a high risk of the virus to their health. They viewed it as serious and anticipated getting infected if they left their residence. They undertook the necessary precautions to prevent infection such as using sanitizer and washing their hands regularly and wearing face masks. They also reported practicing social distancing.

“*...From my perception I think this Corona issue is serious and that's why I have sanitizer in my house, washing my hands and I ensure I wear a mask whenever I leave the house…” (Respondent #38)*
“*...According to me it is serious. I don't leave the house to go anywhere, because if I go out, I might meet anyone and contract the disease and infect my baby. So, I just sit in the house and take care of the baby, maybe I send someone to get me something…” (Respondent #40)*

In extreme cases women, were aware that the COVID-19 carried a risk of mortality if precautions were not taken and one was in a crowded setting.

“*...Coronavirus can cause death if you do not take care of yourself. If you do not take preventative measures when you leave home or are in a crowded place, you can easily get coronavirus…” (Respondent #16)*

On the other hand, a majority of women perceived a low threat of infection to friends and family with 68% mentioned that they perceived their friends and family at a low risk of getting infected by COVID-19. They attributed this to their friends and family being careful and following Government measures to contain the virus.

“*…If you look at my friends, they are really careful. They are trying their best to follow all the rules put in place so that they do not get sick. Then if you look at my family, they are also careful. I do not think anyone will get it unless…for this person who is always going out. But so far I feel they are all being careful and following the rules…” (Respondent#36)*

They described them as taking precautions such as handwashing, sanitizing and wearing of masks when out in the public. These measures led them to believe that most of their friends and family were at a low risk of infection

“…*I don't think they might get infected because I have seen the way they take steps and precautions to prevent that. They wash their hands and sanitize regularly and they also take their clothes off whenever they come from out. So, I cannot say they can get the disease because all of them have masks …” (Respondent #40)*

Almost half of the women perceived a low threat of infection with the virus within their residences. They attribute this low perceived threat to not knowing anyone who had contracted the virus in their neighborhood

“*… I have heard that this disease is killing a lot of people, but I have not heard about anybody contracting this disease in my area.” (Respondent #16)*
*I don't think it is that serious because so far, I haven't heard of any coronavirus confirmed cases here at our place and the residents are observing all the preventive measures put in place by the government through the Ministry of health. (Respondent #43)*

### Perceived Quality of Health Services

The respondents reported varying levels of perceived change in access and quality of services provided as a result of measures put in place due to COVID-19. About half of the women (51%) indicated that they were accessing health services normally and would continue to go to the health facility despite the risk of exposure to the virus.

“*...I haven't changed the way I access the health care services. I'm just going the way I used to, and also as instructed by the nurse in charge of my baby, although I am fearful and worried of who I might meet at the health facility…” (Respondent# 23)*

Respondents perceived quality of care to have improved due to hygiene, caution, attentiveness, privacy, low patient-load, restricted movements, increased numbers of nurses, and shorter turn-around time for patient attendance.

“*…I will say that the quality of service delivered has changed for the better because the Health workers are more vigilant when attending to the patients unlike before when they weren't that much cautious such as observing good hygiene all the time…” (Respondent #41) “…I will also say that the social distancing rule put in place whereby each patient is attended to one by one has made the patients to be more free to the doctors and tell them what they are really undergoing through because in the past, patients could be congested in one room and make the other patient fear to speak up what he or she is going through…” (Respondent #44)*

The change in the health facility culture was perceived to be contributing to improved quality of care. There was an observed change in how COVID-19 preventative measures were enforced by the security at the entrance of the health facility as well as by health workers such as doctors and nurses.

“*...I will say that the quality of service has changed for the better because even the watch man and every health worker is observing all the hygienic measures put in place thus the patients feel safer …they wear masks, protective clothes which they were not using before” (Respondent #45)*
“*...Yes there has been a change in the quality of service e delivered because the health workers are more cautious when handling patients and also the patients are attended to one by one unlike before where three patients could congest in one room....and the way they handle patients is totally different because they handle shifts to prevent overcrowding” (Respondent #46)*

Although most facilities were strict in enforcing the COVID-19 mitigation measures, some respondents reported that experiences at the health facilities they attended as not taking seriously the mitigation measures against COVID-19

“*…they just kept telling us to have our masks on, but it was not that serious. They were not taking this Corona thing seriously. But in facility X you always had to have your mask on. For me I did not see anything different in the facility Y….” (Respondent #36)*

Perceptions of time taken at the facility were mixed. Women described health services that were delivered efficiently at times, and slower health services at other times. Less time was attributed to fewer patients as well as hastened health services.

“*...when I left my home for the hospital, the services were quicker than before because during this COVID-19 period there were fewer patients at the health facility…. the duration taken in the facility was very little and the nurses were fast in their services…” (Respondent #19)*

Although most respondents seemed pleased with the change in the quality of services, some mentioned changes to routine prenatal services. One woman who had taken her baby for immunization witnessed other women who had taken their babies for growth monitoring been turned away by the health care workers. She overheard them been informed that it was unnecessary during the COVID-19 times**.**

“*... I visited the hospital when I took my baby for immunization; at the hospital I observed mothers whose babies were to be weighed were turned back by the nurses who said it is not necessary at this time of COVID-19 they were avoiding crowds…” (Respondent #15)*

Other women also described been turned back because of the large number of people being served

“*...It is because sometimes you can't come there at the hospital because you will not even be served. You see people are many so you go and you are told to go and come back some other day…” (Respondent #41)*

On the other hand, COVID-19 transmission mitigation strategies such as mandatory temperature checks and sanitization at every station were perceived to increase time spent at the facility. The respondents attributed the long waiting time due to health personnel constantly changing their protective gears.

“*…. Before we just used to enter, they write something for you, you go somewhere else but now you see you must be tested first then you go sanitize before you now go inside the hospital…” (Respondent #24)*

Although COVID-19 mitigation strategies had led to perceived improvements in quality at the outpatient department, the inpatient services seemed to have remained the same. There were also incidences of slower services and overcrowding public maternity hospital wards.

“*...That one was tough, because there were many people. When I went to the ward, if any one of us in there would have been having corona, all of us would have contracted it. One bed three people, there's no space to turn, that was a challenge, because those giving birth were many, those undergoing CS were many. There was nowhere to rest, you are just told to go to room three. where you ask for some space on a bed to put your baby, then you can sit on the bench after undergoing the CS. It was quite a challenge….” (Respondent #40)*

### Fear of Infection With COVID-19

While nearly half reported that they were able to access the health facilities, slightly less than half (40%) were hesitant to visit health facilities due to fear, they indicated that their main reason for not going into a health facility was fear. They described uncertainty regarding meeting people with COVID-19 at the health facility and risking exposure to the virus.

“*...I'm going less times compared to non-COVID-19 times. First of all it is because of fear, I'm worried that maybe if I go to the Hospital more often I may get exposed to the people who are infected with coronavirus and unluckily I get infected with the disease too..I am also afraid of exposing my child to the Corona virus when I frequently visit the hospital...” (Respondent #43)*

They further expressed fear of contracting COVID- 19 from health care workers who were regularly being exposed and risked infecting them.

“*... I will be afraid because I don't know if the nurse or the doctor, I find at the facility has the disease. So, I will be afraid…” (Respondent #2)*

This fear of the health facility resulted into some women foregoing scheduled visits to the health facility

“*...It has reduced. I go a few times now. I cannot say it is because of money for me. It is just because I fear to get this disease. It is not easy for me to go to hospital because I fear. I would just stay in the house and wait to feel better. I would just take medicine until I feel better…” (Respondent #36)*

Some respondents also feared to be deemed to be COVID positive if captured by the temperature screening processes that could find their temperature to be high.

“*...You know right now, you do not even want to go to hospital, even when the baby is sick you become scared. You see how they say when you have corona your temperature is high; they might say that you have corona …” (Respondent #36)*

Some of the mitigation measures put in place for COVID-19 were reported to instill fear among community members and the employers of casual laborers which could easily resort to stigma. This stigma resulted into unemployment for some people.

“*...Keeping distance has also made people fear each other…...employers are afraid that you might infect them with COVID-19.” (Respondent #33)*

### Economic and Food Security Challenges

Some women identified economic reasons for not accessing health services. They said that they were unemployed and loss their sources of income and lacked money for transportation to health facilities. They therefore prioritized essential provisions such as purchasing food over going to the health facility.

“*.... As a result of COVID 19. I don't have a job, so the little that we get we prioritize for food then health will come after.” (Respondent #43)*

For those who had some money to purchase food items, they found them to be very expensive because the supply was low.

“*…. you will go to the shop especially to the groceries and they tell you this curfew and lockdown has affected the supply chain and food stuffs are overpriced…” (Respondent #6)*

Reports of food insecurity were also reported as a result of loss of income.

“...*So, there is low income.... sometimes we don't consume the way we used to, we have to minimize the expenditure on the house, food, getting food and something to eat is a challenge …” (Respondent #6)*

The lockdown together with the curfew that was initially put at 7.00 p.m. affected businesses with most of them closing as early as 5.00 p.m. This resulted in a significant loss of gainful income.

“*...Things have become difficult… because going for stuff to come and sell is an issue. I have been staying in the house, I have spent all the money, it is over. If it is time to close work, I have told you I just sell vegetables so these hours of leaving have become stressful. You must close early before 7.00 p.m. Things have become difficult now…” (Respondent #22)*

These difficult economic situations resulted in challenges with psychosocial well-being.

“*...Stress, stress is there because of these lockdowns because business is not there and there is no money and you see I now have 4 children…” (Respondent #5)*
“*...Am stressed with the children, I am the one is blamed by my husband if anything goes wrong in the house, I am also stressed when my baby doesn't get enough milk to breastfeed, all this is happening because of the lockdown and curfew*…”

Children being at home was mentioned as a challenge.

“... *the challenge that I face is that all my children are back at home and none of them is in school…” (Respondent #62)*

The Government initiated curfews to reinforce social distancing not only constrained access to health care services but also led women to attend health facilities unaccompanied. Reports of support companions being denied entry at the health facilities when women sought delivery services.

“*...when my labor started, I went to the hospital x with my cousin and neighbor but could not be allowed in. Unfortunately, this was way past curfew hours (7 pm). However, they couldn't go back to the home and had to stay at the hospital. I had to pretend that I couldn't walk so that the watchman could allow them to help me walk back to the hospital building. After I went inside the maternity they were not allowed inside.” (Respondent #40)*

### Coping With the Challenges Brought About COVID-19

Following the challenges described above, several coping strategies were reported by the respondents. Most of the strategies came from non-governmental organizations and the local administration to cope with loss of livelihood. Non-governmental organizations commenced food distribution to the affected families. In one of the settlements, respondents reported receiving food rations twice a week.

“*...There were people getting aid at the centre A. They were receiving assorted food items. So, for someone like me with a child and I have a very big demand, I queue and then get some food stuff. They come twice a week…” (Respondent #66)*

Other non-governmental organizations distributed items such as soap and money

“*...one day we were given a bar of soap with an organization called H”... “I have received a little support in terms of funds and am so thankful for that.” (Respondent # 035)*

Some respondents however mentioned favoritism in food distribution, citing a lack of transparency and clear criteria.

“*...Have not seen anything or maybe it's given and am not aware because I hear from people food is distributed at night to those who are known to the people concerned…” (Respondent # 62)*

However, the support received from the local administration fell below community members' expectations. They expected economic support, but the support they received was often advisory and related to COVID-19 containment measures. The area administration and landlords were reported to have teamed up to enforce curfew measures.

“*...The Member of County Assembly (MCA), and the Chief, even the landlords have also helped when it came to warning and enforcing the restrictions. He used to call and warn people about letting strangers in and encouraging people to report anyone who does it*…*they should give food to people like us who can't go to work because I wash clothes for people but now, they can't allow us in the house. Food is very expensive at the moment people are taking advantage by adding the prices of items. Because you know food is the reason you will go get infected with these diseases because you are going look for food.” (Respondent #24)*

### Recommendations by Women to the Government

Our respondents were mostly concerned about food, basic supplies, rent. and financial assistance. They made no recommendations regarding their access to health services. The most important recommendation was for the Government to provide food with one third of the women recommending this. They suggested that the Government should support families economically by providing food items so that people don't have to go look for work and thus endanger their families.

“*… providing essential needs such as food...talking to the landlords at least yeah the rent issue is also a problem for so many of us… If they can get someone to help us with pads because there are no pads.” (Respondent # 6)*

In addition, some saw the role of the Government as going beyond food items to encompass provision of hand sanitizers and masks; as well as ensuring that families were not evicted from their houses due to lack of rent.

“*...The government should buy for people mask and sanitizer, there is no money paying for house rent is a problem the government should talk to the owners of the house to make them understand what people are going through, those people who work their time is limited because of curfew…” (Respondent # 65)*

## Discussion

Our study sought to explore the maternity experiences women residing in informal settlements during the COVID-19 pandemic. Specifically, we assessed women's knowledge of COVID-19, their perceived risk of infection, access to health facilities, perceived quality of care and challenges experienced as a result of the COVID-19 pandemic mitigation measures. Overall, the findings revealed important effects of the pandemic on maternity care and access, suggesting some improvements in quality of care as well as some continued challenges with access and quality that were further exacerbated by the pandemic. In addition to their reflections on maternity care, specifically, women also commonly reported more general concerns and economic stressors about the effects of the pandemic that have important implications for how multisystemic supports might be put into place to support perinatal women.

The findings revealed that women continued to access health facilities and would continue to do so despite the perceived risks of infection by COVID-19. Less than half of women reported reduced access due to fear of contracting the coronavirus, deprioritization of health services, economic constraints, psychosocial effects due to the imposed lockdown and curfews. Some of the reduced access was due to new hospital policies that restricted women's entry to the health facility. These changes resulted in hospital policies that dictated for certain routine services such as growth monitoring were unimportant during this time due to the risk of contracting COVID-19. Other restrictions included prohibition of friends and family accompanying women to the health facility at a time when they were needed most by the women. Jolivet et al. (24) emphasizes that respectful care and human rights need to be upheld even at times of a pandemic such as the COVID-19. They call for the use of ethical guidelines in access to proven practices during maternity care.

Most of the respondents perceived improvements in quality of care due to short-waiting times, hygiene measures and responsive health personnel. However, this was only reported for the outpatient services as some in-patient services remained overcrowded. While the Government measures to contain COVID-19 were lauded and appreciated by all, there were several effects; some were unintended. This includes fear of exposure to the virus, economic constraints and effects on psychosocial well-being. While nearly half reported that they were able to access the health facilities, slightly less than half (40%) were hesitant to visit health facilities due to fear, stigma, and reported lack of proper COVID-19 preventive measures at the facilities. This is in line with findings of a study in West Africa conducted during the Ebola outbreak that illustrated that the outbreak disrupted services and fear of seeking treatment (2). There were reports of overcrowding especially in the inpatient department and most mothers were scared due to lack of physical distancing which is one of the measures for preventing COVID-19 infections

Women's positive response to the increased attention and privacy their received during care highlight that the COVID pandemic mitigation measures at health facilities might have led to better experiences of patient centered maternity care and dignified care particularly during outpatient care. Studies focused on women experiences during facility-based delivery highlight High quality patient centered maternity care as represented by dignified care including privacy (25).

Our findings also illustrate that awareness of the symptoms and preventative measures for COVID-19 was high. These findings are consistent with a study by the Population Council to assess COVID-19 knowledge attitude and practices in informal settlement in Nairobi showed that knowledge on COVID-19 symptoms was high and respondents could name several preventative methods (14). Our findings however differ from other studies conducted by White Ribbon Alliance (WRA) in selected counties in Kenya between April and May 2020 to determine the impact of COVID-19 on reproductive, maternal, and newborn health services. Whereas our study reported high levels of knowledge on COVID-19, this study found out that many citizens who live in slums in other parts of Kenya, did not know about the curfew. This was attributed to women lacking phones or radios, and the fact that information takes time to disseminate particularly in rural areas. This might also possibly be due to the changing demographics that indicate that women in informal settings are increasingly younger and possess secondary education (26). Our study was also conducted 2 months later which could possibly explain the learning curve. Findings show that women's perception of risk to themselves was high, but that perception of risk to family and friends, and in their neighborhood was low.

A majority of the participants had lost their jobs and their source of livelihoods and were struggling with access to income. Consequently, there were reports of lack of food, lack of rent and stress. To cope with these challenges, some women reported receiving support from the non-governmental organizations as well as government local administration. However, they reported that government support was focused on enforcing COVID-19 preventative measures while women's priority was food and rent. Our findings conform with other studies that have been conducted during the COVID-pandemic that indicate that effects of the lockdown have been primarily economic (14). In the case of women's access to maternity services, the primary access difficulty may therefore be women's in ability to pay for costs associated with accessing health care, despite its ongoing availability and women's overall willingness to use it. Evidence from the Ebola virus outbreak in 2013–2016 in Western Africa shows the negative indirect effects that such crises can have on sexual and reproductive health (2).

A major cross cutting issue from our study was stress related to the loss of livelihoods and severe economic constraints. Women reported being stressed due to unemployment, food security, rent, and sudden school closures. This economic hardship led to some women reducing their access to health care by prioritizing their finances to basic provisions as opposed to using it for transportation costs to the health facility thus reducing access to health services overall. We recommend that state and non-state actors should focus efforts on the impacts of income loss and food security, with special attention to women. This can be achieved by ensuring that those most at need of assistance are the ones receiving it by considering that assistance is getting into the hands of women given their increased experience of social and economic impacts.

A major limitation of the study was that the first lock-down which had restrictions on movement after 7 p.m. and before 4.00 a.m. greatly limited travel. The only plausible means to communicate effectively with the women was via mobile phones. The penetration of mobile phones is reasonably high within the urban slums of Nairobi. However, this might have limited the population we interviewed to women who are slightly economically stronger because of the ability to afford and use a mobile phone.

## Conclusion

It is likely that despite the best efforts of health professionals, that an upward surge in the numbers of COVID-19 related deaths in women of reproductive age, including pregnant and post-natal women, will take place. It is crucial, however, to continue every effort as a vital contribution to safe childbirth and high-quality maternal care, and to continue to work toward the achievement of sustainable development goals. Although the Kenyan Ministry of Health (MOH) launched a COVID-19 Taskforce to steer the country's prevention, containment and mitigation measures, there is need to include additional measures to prevent the devastating health, social and economic impact of a COVID-19 outbreak particularly among women living in informal settlements.

## Data Availability Statement

The raw data supporting the conclusions of this article will be made available by the authors, without undue reservation.

## Ethics Statement

The studies involving human participants were reviewed and approved by Strathmore University IRB Ethics Committe. The patients/participants provided their written informed consent to participate in this study.

## Author Contributions

JO-A, LM-G, and RD conceptualized the study. JO-A, TC, CM, and MN analysed the data. JO-A, TC, and MN wrote the manuscript. JO, DG, and LM-G provided critical feedback to the manuscript. All authors read and approved the manuscript prior to publication.

## Conflict of Interest

The authors declare that the research was conducted in the absence of any commercial or financial relationships that could be construed as a potential conflict of interest.
