# Supplementary material for: COVID-19 Effect on Access to Maternal Health Services in Kenya
Source: Front Glob Womens Health. 2020 Nov 26;1:599267. doi: 10.3389/fgwh.2020.599267 (PMC8593959; doi:10.3389/fgwh.2020.599267)
Supplement: Supplementary file 1 [file Table_1.DOCX]

**Appendix I : In-depth Interview Questionnaire**

**Topic: Is the imposed COVID-19 lockdown reducing women’s access to maternal health services within informal settlements in Nairobi, Kenya?**

| 1. Have you heard about covid-19, also known as the novel coronavirus? What do you know about it? |
| --- |
| 1. How serious do you think the Covid-19 outbreak is for you in your neighborhood? 2. Of your family members and friends, how many do you think will get sick with the coronavirus? |
| 1. For an average person in good health, how serious a threat does the coronavirus pose to your health? 2. Have you changed the way you access health care services for routine health care services such as child welfare services postnatal care services or routine health care services during the outbreak? If so how? If you have changed are you going more times or less times and if so, what are the reasons? PROBE; economic? Fears? 3. If you got sick during the COVID-19, do you think you will go to the hospital? 4. Have you gone to the health facility during the COVID-19? Were you treated differently? Were there any different procedures? If so, what are they doing that is different? 5. Was the health facility doing any of the following hand washing? use of sanitizers? Measuring the temperatures? Isolation wards? Any Others things that you have observed that are different during the non-COVID times? 6. When at the health facility what are the practices that are been adopted by health care workers such as taking precautions such as donning personal protective equipment leading to more waiting time or a different quality of service? If yes please explain? 7. The government has put strong measures in place to discourage people from leaving their homes- a lockdown and a curfew. Overall, can you describe your experience during the lockdown during COVID 19? Have you experienced good health or bad health? Are you experiencing any health disorders such as depression? are you experiencing violence from your partner or frustrations? Please elaborate? 8. What practices are your community putting in place to prevent the spread of the COVID-19? 9. What kind of support are you receiving from within your community or from the Government to deal with the COVID-19 outbreak? 10. Who particularly has shown leadership in dealing with the COVID -19 within your community? 11. What are the challenges that you are facing in the light of the Government directives aimed at reducing the spread of COVID -19? 12. What would you like the Government to do to reduce the spread and economic impact and COVID -19 in your community? |
|  |
|  |
|  |
