# Supplementary material for: COVID-19 Effect on Access to Maternal Health Services in Kenya
Source: Front Glob Womens Health. 2020 Nov 26;1:599267. doi: 10.3389/fgwh.2020.599267 (PMC8593959; doi:10.3389/fgwh.2020.599267)
Supplement: Supplementary file 2 [file Table_2.DOCX]

**Appendix II**

**Open Codes**

| **Text** | **Open code** | **Sub theme** | **Theme** |
| --- | --- | --- | --- |
| “For me the disease is not that much serious because according to the  information that I have is that the disease is more dangerous to the people who are already  suffering from another disease because their immunity is low.” | The disease is not that much serious; information that I have is that the disease is more dangerous to the people who are already  suffering from another disease | Awareness of disease; Low risk perception to one’s health | Awareness and risk perception on COVID 19 |
| “Personally I’m seriously threatened with disease because I know in case I get  infected the chances for me to survive are very low.” | I’m seriously threatened with disease; I know in case I get  infected the chances for me to survive are very low | Awareness of disease; High risk perception to one’s health | Awareness and risk perception on COVID 19 |
| “Not that serious because all I know is that the people who are infected with the other diseases are the ones at a higher risk of encountering death quickly which is different to my case because I believe that I will survive in case I get infected since I haven’t had any other health complications.” | all I know is that the people who are infected with the other diseases are the ones at a higher risk of encountering death quickly; I believe that I will survive in case I get infected since I haven’t had any other health complications | Awareness of disease; Low risk perception to one’s health | Awareness and risk perception on COVID 19 |
| “I will say neither my family members nor my friends get sick of coronavirus because they are observing all the measures put in place in order to prevent the spread of coronavirus.” | neither my family members nor my friends get sick of coronavirus; they are observing all the measures put in place | Awareness of disease; low risk perception of infection to family or friends | Awareness and risk perception on COVID 19 |
| “...I haven’t changed the way I access the health care services. I’m just going the way I used to,  and also as instructed by the nurse in charge of my baby, although I am fearful and worried of who I might meet at the health facility” | I haven’t changed the way I access the health care services; although I am fearful and worried of who I might meet at the health facility | Same routine healthcare services | Perceived quality of health services |
| “It has changed because like now you can’t go to take the baby to measure their  weight, you only take them for injection. If it is weight you are told to relax at home.” | like now you can’t go to take the baby to measure their  weight; you only take them for injection | Reduced visits to health facility | Perceived quality of health services |
| “I will say that the quality of service delivered has changed for the better because the Health workers are more vigilant when attending to the patients unlike before when they weren’t that much cautious such as observing good hygiene all the time.” | the quality of service delivered has changed for the better; Health workers are more vigilant when attending to the patients unlike before when they weren’t that much cautious such as observing good hygiene | Improved health service delivery | Perceived quality of health services |
| “…I will also say that the social distancing rule put in place whereby each patient is attended to one by one has made the patients to be more free to the doctors and tell them what they are really undergoing through because in the past, patients could be congested in one room and make the other patient fear to speak up what he or she is going through” | the social distancing rule put in place whereby each patient is attended to one by one has made the patients to be more free to the doctors and tell them what they are really undergoing; in the past, patients could be congested in one room and make the other patient fear to speak up | Improved health service delivery | Perceived quality of health services |
| “When I left my home for the hospital, the services were quicker than before because during this COVID-19 period there were fewer patients at the health facility….the duration taken in the facility was very little and the nurses were fast in their services.” | the services were quicker than before because during this COVID-19 period there were fewer patients at the health facility; .the duration taken in the facility was very little and the nurses were fast in their service | Improved health service delivery | Perceived quality of health services |
| “They call one mother  and her child at a time which leads to more waiting time” | They call one mother  and her child at a time which leads to more waiting time | Slow service delivery | Perceived quality of health services |
| “ I was forced to close down business because there are no customers.” | forced to close down business because there are no customers | Closed down business | Psychosocial economic challenges |
| “My husband works in construction and at times he has been called for jobs outside of Nairobi and as a result of the lock down he can’t go. This has affected us financially.” | at times he has been called for jobs outside of Nairobi and as a result of the lock down he can’t go; This has affected us financially | Restricted movement due to lock down; no finances | Psychosocial economic challenges |
| “You know someone comes and quarrels with you and maybe they beat you and they know there is nowhere you will go because there is lock down. And if it wasn't there if you say go once they leave you go. But now there is lockdown, where will you go? You just have to persevere and stay there.” | someone comes and quarrels with you and maybe they beat you; they know there is nowhere you will go because there is lock down; You just have to persevere and stay there. | Disagreements with partner | Psychosocial economic challenges |
| “This lockdown has made it so that you can't visit each other.” | This lockdown has made it so that you can't visit each other | No social interaction | Psychosocial economic challenges |
| As a result of COVID 19. I don’t have a job, so the little that we get we prioritize for food then health will come after. | As a result of COVID 19. I don’t have a job; the little that we get we prioritize for food then health | Loss of jobs | Psychosocial economic challenges |
| “...Stress, stress is there because of these lockdowns because business is not there and there is no money and you see I now have 4 children… | Stress, stress is there because of these lockdowns; business is not there and there is no money | Stress and lack of money | Psychosocial economic challenges |
| “One day we were given a bar of soap with an organization called H” ... I have received a little support in terms of funds and am so thankful for that..” | we were given a bar of soap with an organization called H; I have received a little support in terms of funds | Received support from an organization | Mitigation strategies |
| “The Member of County Assembly (MCA) , and the Chief, even the landlords have also helped when it came to warning and enforcing the restrictions. He used to call and warn people about letting strangers in and encouraging people to report anyone who does it.” | The Member of County Assembly (MCA) , and the Chief, even the landlords have also helped when it came to warning and enforcing the restrictions; warn people about letting strangers in and encouraging people to report anyone who does it | Support from MCA, Chief and Landlords with enforcing restrictions | Mitigation strategies |
| “But I think once, I heard people out there saying that the government, people came with sanitizers saying that they were from the Government.” | people came with sanitizers saying that they were from the Government. | Received support from the government | Mitigation strategies |
| “My elder sister keeps on reminding the community members to observe the safety  measures and also telling them not to look down upon COVID-19 because the disease is real  and it is killing people.” | My elder sister keeps on reminding the community members to observe the safety measures | Sensitization from civilians within the community | Mitigation strategies |
| “What I know every  business in the community have put a place where there customers wash they hands before and after  purchasing.” | every  business in the community have put a place where there customers wash they hands before and after  purchasing | Initiative by business people in the community | Mitigation strategies |
