# Supplementary material for: COVID-19 Effect on Access to Maternal Health Services in Kenya
Source: Front Glob Womens Health. 2020 Nov 26;1:599267. doi: 10.3389/fgwh.2020.599267 (PMC8593959; doi:10.3389/fgwh.2020.599267)
Supplement: Supplementary file 3 [file Table_3.DOCX]

**Appendix III**

**CODING FRAMEWORK AND FREQUENCIES FOR COVID INTERVIEWS**

**THEME 1: GENERAL KNOWLEDGE**

| **Respondent ID** | **Knowledge on COVID (Symptoms)- high/ low** | **Knowledge on Preventive measures- high/low** | **Perceived risk of infection**  **(one’s area of residence)** | **Perceived risk of infection (family/ friends)** | **Perceived threat to one’s health** |
| --- | --- | --- | --- | --- | --- |
| 1 | High | N/A | High | Low- are observing measures | High |
| 2 | Low | Low | High | Low-for family | High- I don't know how my immunity is if it's low. |
| 3 | Low | Low | High | Low- are observing measures | N/A |
| 4 | Low | High | Low- has not heard of anyone infected | Low | Low- Me I don't see as if it can affect me. Because I don't have any illness, I haven't fallen sick anywhere. |
| 5 | Low | High | High | Low | High- is diabetic |
| 6 | High | N/A | High | Low | High |
| 7 | High | High | High | High- They are at a higher risk because at first they were observing preventive  measures but right now they are very reluctant where by you just see them walking around  without wearing masks | Low- For me the disease is not that much serious because according to the  information that I have is that the disease is more dangerous to the people who are already  suffering from another disease because their immunity is low. |
| 8 | High | High | Low | Low | High |
| 9 | High | High | Low | Low | High- Personally I’m seriously threatened with disease because I know in case I get  infected the chances for me to survive are very low. |
| 10 | High | High | High- already have 5 confirmed cases in the area | Low | High- fears infecting baby |
| 11 | High | High | Low | Low | High |
| 12 | High | High | High | Low | High |
| 13 | High | High | High | Low | N/A |
| 14 | High | N/A | Low- Not that serious because I haven’t witnessed any coronavirus case here at  Dandora. | Low | High |
| 15 | High | High | N/A | Low | N/A |
| 16 | High | High | N/A | High | High |
| 17 | High | Low | Low | Low | N/A |
| 18 | High | High | Low | Low | Low  -Not that serious because all I know is that the people who are infected with  the other diseases are the ones at a higher risk of encountering death quickly which is  different to my case because I believe that I will survive in case I get infected since I haven’t  had any other health complications. |
| 19 | High | High | Low -not seen anyone infected | Low | High  -serious disease |
| 20 | Low | Low | Low -no infected person | Low | Low-  I don’t think I have to worry over coronavirus because my health is okay and  my practicing the preventive measures stated by the Ministry of Health. |
| 21 | Low | Low | High -are already has 7 confirmed cases | Low | High |
| 22 | High | High | Low | Low | Low |
| 23 | Low | Low | Low | Low | High |
| 24 | High | Low | High | Low | High |
| 25 | Low | Low | Low | Low | Low |
| 26 | High | Low | High  its crowded keeping distance is not easy and also clean water is a big problem  because we buy. | N/A | N/A |
| 27 | High | High | High | High | High  It can pose a threat, because sometimes when I go to the hospital, I  am usually told I have pressure. |
| 28 | High | High | High | Low | High |
| 29 | High | High | High | High | Low  I see no threat to my health as long as I do as the government as told us; |
| 30 | High | High | Low | High | High |
| 31 | Low | Low | Low | N/A | N/A |
| 32 | Low | High | N/A | Low | High |
| 33 | Low | Low | Low | Low | High |
| 34 | High | High | Low | Low | Low |
| 35 | High | Low | Low | High | N/A |
| 36 | Low | High | High | Low - if you look at my friends they are really careful Then if you look at my family, they are also careful. | Low |
| 37 | High | High | Low | High - You know like my husband can get the disease from outside then spread it to the rest of the family likewise friends. | Low |
| 38 | High | High- From my perception I think this Corona issue is serious and that’s why I have sanitizer in my house, washing my hands and I ensure I wear mask whenever I leave the house. | Low | Low | High - For me it’s serious because through watching TV I see the cases increasing each day that I don’t think if it will end any soon |
| 39 | Low | High - we have been told to keep social distance, wash our hands regularly and in case you feel unwell there is a number to be called. | Low - Have not heard of any case in this area. | Low | Low - unsure of the risk for an average person. |
| 40 | High | High - And whenever you come from out, you must always wash your hands, and if you have travelled far you must remove your clothes and shower first before doing anything els | High- fear of contracting disease and infecting the baby. | Low - they practice preventive measures | N/A |
| 41 | Low | High | High - economically. right now people are crying there are no jobs even by getting one for a day you just thank God. | N/A, - didn't answer the question on risk. | High - it is a bad this getting that disease when you have a small baby like this that will send my life to be completely finished. |
| 42 | Low | High | High - peopel are wearing masks. | High- Yes, they can be affected with covid-19 more so those who don’t follow instructions like avoiding crowded places, failing to wear masks in the meetings and failing to sanitize. | Low - unaware of the risks. |
| 43 | Low | High | Low - haven't heard of any cases. | Low - Because all my family members are observing all the preventive measures against coronavirus | High - Very serious because according to the information received from the Ministry is that the people who are infected with another disease are likely to die when they get sick of this disease which I find it so threatening to my side because I’m suffering from Diabetes lately. |
| 44 | High | High | High - inspite of the lack of cases in the area. | Low- I will say neither my family members nor my friends get sick of coronavirus because they are observing all the measures put in place in order to prevent the spread of coronavirus. | Low - not that serious because I’m observing all the preventive measures wherever I am so I will say it isn’t a threat to me that much. |
| 45 | High - | High | Low- because people are not observing preventive measures. I will say that it isn’t that serious because some of the residents aren’t following the instructions put in place by the government such as wearing face masks most of the people aren’t observing the rule.  - | High - family. Partner is not cautious. He is not observing all the preventive measures put in place by the government such that he is always in a group and not maintaining social distance when interacting with other people. | Low - does not have any pre-existing conditions. |
| 46 | High | High | Low - I will say that the disease isn’t that much serious because I haven’t heard of any confirmed coronavirus cases even though not many people have undergone the testing. | Low - they are following the preventive measures | Low - It won’t be serious to my health. I just see it the same as other diseases like a common cold so in case I am infected I know I will survive.  Interviewer: Have you changed the way you |
| 47 | High | High | Low - it’s not that serious because I have only heard of one confirmed case so far but the patient recovered. | Low - observing prevention measures. | High - Coronavirus is a deadly disease and I’m fully worried about this because if I get I might die |
| 48 | Low - knowledge of how the disease spreads but not symptoms. | High | High | High - if they don't take precautions | High -especially if they have a small baby. It is a threat to me because I'm having a small baby |
| 49 | High | High | Low | Low | High - if person has pre-existing health conditions. |
| 50 | High | High | Low - unaware of the risks as they are taking precautions. | Low | Low - Except if one has poor health/pre-existing health conditions. |
| 51 | Low | Low | High | N/A | Low - unless they have poor health. Someone with good health it can overwhelm them a little but not as much as someone with bad health. |
| 52 | High | Low | High-due to congestion  You know where I am staying, we are so congested. So there it is very serious because you can easily get it. | Low - You know now there, because we are far from each other we cant… It's hard. | High - You know now if it gets one person it must spread to everyone. So that is dangerous to the family. |
| 53 | High | Low | Low - Here I have not heard of these cases and even if it's there, maybe people maybe have recovered. | Low | Low - unless it is someone with weak immune system |
| 54 | Low | High | Low | N/A | High - especially for someone with a small baby. It can be serious when it gets you because you see someone like me, I am in the house with children, I am breastfeeding and I have a small baby, it can be serious. Because I also heard that young infants weeks old are getting it. |
| 55 | High | High | Low | N/A | High - When you touch dirt, is when you get the disease. Also, if you are near someone who has it you will get it. |
| 56 | High | Low | Low - Its not that serious. I have seen anyone who has gotten it. | Low | High - let's say if you have another disease now there it might really affect you. |
| 57 | High | High | Low - Here we have not seen it… We are just protecting ourselves. | N/A | High - But I heard this disease fights with the body so if your body is strong it can fight with it and you recover and if you are weak, and then let's say if you have a disease like diabetes, it can kill you also, cancer, hiv… Things like that. |
| 58 | Low | Low | Low - can't perceive risk. | Low | High - Each and every day people are getting the virus more people are dying am worried anything can happen you will never know you have to keep yourself safe. |
| 59 | Low | High | High - The disease is serious people are affected and people can’t travel people don’t go to work and live is hard. People are suffering me being one of them. | Low | Low - unable to determine threat. |
| 60 | High | High | High - Where I stay it is crowded and people can’t observe to be one meter apart it’s not easy in any case the disease can be where I live everybody will be affected with you like it or not. | Low - For now it is not easy to know most of them have not done the test so people don’t know their status and that is why we are told to stay home stay safe. | High - We are told that everyone can get corona virus |
| 61 | N/A | High | Low | Low | Low |
| 62 | N/A | High | High | Low | High |
| 63 | Low | High | High | N/A | High |
| 64 | Low | Low | N/A | N/A | High |
| 65 | Low | N/A | N/A | N/A | N/A |
| 66 | N/A | N/A | High | High  -My family members are ignoring the rules. Mostly my friends are the ones who are not obeying the rules, they don’t want to keep social distance, wearing of masks is a problem | Low  I think I will survive if I ever get corona. |
| 67 | High | High | High  -In Dandora, quite a number of people are not following the rules and precautions. | Low | Low  I have faith that if I ever get it, I will recover without too many problems. |
| 68 | High | High | High | Low | High |
| 69 | High | High | Low | High | High |
| 70 | High | High | High | Low | N/A |
| 71 | High | High | High | High | High |
| FREQUENCY | HIGH- 44 **(62%)**  LOW- 24 **(34%)**  N/A- 3 **(4%)** | HIGH- 44 **(62%)**  LOW-22 **(31%)**  N/A- 5 **(7%)** | HIGH- 31 **(44%)**  LOW- 35 **(49%)**  N/A- 5  **(7%)** | HIGH- 13 **(18%)**  LOW- 48 **(68%)**  N/A- 10 **(14%)** | HIGH- 40 **(56%)**  LOW- 21 **(30%)**  N/A- 10  **(14%)** |

**THEME 2: ACCESS TO HEALTH CARE SERVICES**

| **Respondent ID** | **Routine healthcare services**  **(same, less, more)** | **Reason- economic, fear etc** | **Future access to healthcare services- yes/ no** |
| --- | --- | --- | --- |
| 1 | Less | economic | No- limited due to financial constraints |
| 2 | Same | N/A | Yes- but I will be afraid because I don't know if the nurse I will get there or the doctor has the disease. So I will be afraid |
| 3 | Same | N/A | Yes |
| 4 | Less | -one is not able to go to the hospital at night (curfew)  -clinics request to take baby for only injections and not weight measurements | Yes |
| 5 | Less | Fear | Yes |
| 6 | Less | Fear- So sometimes you can just even just call a doctor and tell him  you feel this and this and this and this so instead of going there he can even prescribe for you  from the phone. | Yes |
| 7 | Less- Nowadays I don’t go for the health care services more often like I used to  go before the outbreak of coronavirus. | Fear- It is because of fear of not knowing the health condition of the person that I’m  going to meet and the healthcare worker that is going to attend to me or my child. | Yes- I will go because it is my personal health which I should give priority to. |
| 8 | Same | N/A | Yes |
| 9 | Same | N/A | Yes |
| 10 | Same | N/A | Yes |
| 11 | Same | N/A | Yes |
| 12 | Less | N/A | Yes |
| 13 | Same | N/A | Yes |
| 14 | Less | Fear- I will say its fear because going to the Hospital will make me more exposed and  therefore I might be at a higher risk of getting infected with Coronavirus. | No-  This is because going to the hospital I will be exposing myself to the disease and  many people at the Hospital are the one who are likely to be infected so I can’t go. |
| 15 | Same | N/A | Yes  -I will go but very cautious |
| 16 | N/A | N/A | Yes |
| 17 | Same | N/A | Yes |
| 18 | Same | N/A | Yes  -But a private hospital |
| 19 | Same | N/A | Yes |
| 20 | Same | N/A | Yes |
| 21 | Less | Fear  the hospital is where we have all kinds of patients so you don’t know  who you are going to meet and you don’t have any idea about their health condition. | Yes |
| 22 | N/A | N/A | Yes |
| 23 | Less | See now it's like the body has refused. It’s like bodies are not getting sick. Bodies are afraid of corona until all the other diseases have gone far. | Yes |
| 24 | Less | It has changed because like now you can’t go to take the baby to measure their  weight, you only take them for injection. If it is weight you are told to relax at home. | Yes  -You can go if you are serious but if you are not that serious you just relax. |
| 25 | N/A | N/A | Yes |
| 26 | N/A | N/A | No  - I will go to a chemist to buy drugs. |
| 27 | Less | Fear  -At the hospital you interact with so many people of whom you don’t  know if they have the virus or not. | Yes |
| 28 | Same | N/A | Yes |
| 29 | Same | N/A | Yes |
| 30 | Same | N/A | Yes |
| 31 | Same | N/A | Yes |
| 32 | Same | N/A | Yes |
| 33 | Same | N/A | Yes |
| 34 | Same | N/A | Yes |
| 35 | Less | Fear | Yes |
| 36 | Less - but to private.  Because people are not many there. People do not have money and so they cannot afford to go to private hospitals they go to public. Also, private hospitals will not want to spoil their business and so people will not get corona from there. | Fear - You know right know you do not even want to go to hospital, even when the baby is sick you become scared. | No-  It is not easy for me to go to hospital because I fear. I would just stay in the house and wait to feel better. I would just take medicine until I feel better. |
| 37 | Same | N/A | Yes |
| 38 | Same- but taking precautionary measures. | N/A | Yes |
| 39 | Same - but practicing precautionary measures | N/A | Yes - to find out if it's corona |
| 40 | Same | N/A | Yes |
| 41 | Less - as instructed. | N/A | Yes |
| 42 | Same | N/A | Yes |
| 43 | Less | Fear - First of all it is because of fear, I’m worried that maybe if I go to the Hospital more often I may get exposed to the people who are infected with coronavirus and unluckily I get infected with the disease too. | No - might not go because of the financial problems that I have as a result of COVID 19 because I won’t have money to take me to the Hospital. |
| 44 | Same | N/A | Yes |
| 45 | Less | -Fear  -financial constraints | Yes |
| 46 | Same | N/A | Yes |
| 47 | Same | N/A | Yes |
| 48 | N/A | N/A | Yes |
| 49 | Same | N/A | Yes |
| 50 | Same | N/A | Yes |
| 51 | Same | N/A | Yes |
| 52 | Less | Fear - Many you know are afraid. Because before when you went to the clinic you would find many people but now they are not many. | Yes |
| 53 | Less | Precautionary - Turned back for weight taking visits to reduce numbers at the hospital | Yes |
| 54 | Less | Fear - I think it is because of that disease. | Yes |
| 55 | Less | N/A | Yes |
| 56 | Less | Because they are not sick | Yes |
| 57 | Same | N/A | Yes |
| 58 | Same | Economic - Have not changed its only one hospital that am going to Bro – Andre, corona virus you have to be extremely careful about it even if you are going anywhere, the economic as per know if high the lockdown to get someone giving you money like my husband is not around he is in Mombasa its challenging at the moment. | Yes - to confirm if they have the disease. |
| 59 | N/A | N/A | Yes - Yes I can go to the hospital for treatment maybe I might Covid – 19. |
| 60 | Same | Fear- change the mode of transport to the hospital to avoid contact. | Yes |
| 61 | N/A | N/A | Yes |
| 62 | Less | you’re told in advance not to take the baby to the clinic the time of height and weight. | Yes |
| 63 | Less | N/A | Yes |
| 64 | Same | N/A | Yes |
| 65 | N/A | N/A | Yes |
| 66 | Less | Fear  You might find someone having flu and is scared to go to the hospital for fear of being taken to the quarantine center. | Yes |
| 67 | Less | -I am scared of going to the hospital. I am not sure if the doctor treating me is infected with the corona virus.  -They recommended that you should only take the baby there for inoculations and serious illnesses. | Yes |
| 68 | Same | N/A | Yes |
| 69 | Less | Fear  everytime you think of going to the hospital you are afraid because you don't know what you will encounter | Yes  But you can persevere it's not like before someone used to get some slight discomfort you run to the hospital at least now someone just perseveres to see if there is a way you can recover. |
| 70 | Same | N/A | Yes |
| 71 | Less | Fear -I can’t go every time I persevere because I fear.  No finances | Yes  -If it comes and it is serious I will go but if it is not serious I don’t see the need. |
| FREQUENCY | SAME- 36 **(51%)**  LESS- 27 **(38%)**  N/A- 8 **(11%)** |  | YES- 66 **(93%)**  NO- 5 **(7%)** |

**THEME 3: PRACTICES ADOPTED DURING COVID 19 TIMES**

| **Respondent ID** | **Change in healthcare services** | **Effects on quality of care- short waiting time, long waiting time etc** | **Effects on quality of care- health worker behavior, other changes etc** |
| --- | --- | --- | --- |
| 1 | Yes | Long waiting time | Health Workers are more cautious |
| 2 | Yes | N/A | N/A |
| 3 | Yes | Long waiting time | N/A |
| 4 | Yes- hospitals are taking fewer people | Short waiting time | N/A |
| 5 | Yes | Short waiting time- process is long but nurses are ready to attend to you | N/A |
| 6 | Yes | Waiting time still the same | N/A |
| 7 | Yes | Waiting time still the same | N/A |
| 8 | Yes | Shorter time | N/A |
| 9 | Yes | Shorter time | quality has changed positively because the health of the patients is being taken care of such that they can’t feel to be at any risk of being exposed with this COVID-19  period |
| 10 | Yes | Shorter waiting time | N/A |
| 11 | Yes | Long waiting time | Health Workers are more cautious |
| 12 | Yes | Same | N/A |
| 13 | Yes | Same | The quality of service delivered was much better than before because they  observed all the preventive measures and therefore the patient can’t be infected with the  disease. |
| 14 | Yes | Longer waiting time  I will say that the treatment process was much slower compared to non-  COVID times where patients are attended one by one to ensure there are no close contacts  among the patients such as sitting in the same room without observing social distancing. | N/A |
| 15 | Yes | Shorter time | Yes-  The environment is clean and services are very fast. |
| 16 | Yes | N/A | Yes  During this time we go in  the observation room one woman and her child at a time unlike the previous times before  covid-19 when several women could go in the observation room.  -There were fewer people at the clinic compared to  the times before covid-19. |
| 17 | Yes | N/A | N/A |
| 18 | Yes | Same waiting time | Yes  -The quality has changed for the better because all the health workers are  cautious and observing the preventive measures such as putting on gloves and wearing  masks. |
| 19 | Yes | Shorter waiting time  -the services were quicker than before because during this COVID-19 period there are lesser patients at the health facility. | Yes  -Health workers are more vigilant in the way they treat the patients thereby making the patients safer from getting any other disease than before during the non-COVID-19 times. |
| 20 | Yes | Longer waiting time  -The process was too much longer because the health workers take much  time to observe the preventive measures against coronavirus such as washing hands every  time which makes us as the patients to wait much longer. | N/A |
| 21 | Yes | Longer waiting time  -The wearing of gloves among the health workers made the procedure slow  because each time they had to change their gloves after attending to any patient. | N/A |
| 22 | Yes | Longer waiting time | Yes  -The difference that is there is that in terms of treatment, they are treating well.  -they are very strict with cleanliness |
| 23 | Yes | Shorter waiting time- when you arrive you don’t take too long, you  get attended to quickly so that you leave. | N/A |
| 24 | Yes | Longer waiting time  -Yes they made me wait because the services were one by one slowly and carefully no one was getting close to anyone so it had to be done slowly. | -the services were done very fast compared to the time there was no  Coronavirus.  -The nurses had put on gloves and they observed time. |
| 25 | Yes | Shorter waiting time  time of waiting to be served is very short. | -The facility is not busy and crowded the way it used to be before covid 19 |
| 26 | Yes | Shorter waiting time | -What I observed is how time is managed, before you could go to a health facility and wait  for more than 30 minutes for nurses during tea break.  -The service is very fast, |
| 27 | Yes | N/A | -The doctor attends to you but there is that  feeling of him being scared that you might affect him with covid 19. We need the  services and he’s the service provider but there is a lack of trust and friendship |
| 28 | Yes | Longer waiting time  -They call one mother and her child at a  time which leads to more waiting time, unlike before | They keep the clinic clean. |
| 29 | Yes | Longer waiting time | N/A |
| 30 | Yes | Longer waiting time  -They call one mother  and her child at a time which leads to more waiting time, | N/A |
| 31 | Yes | Longer waiting time | N/A |
| 32 | Yes | Same waiting time | N/A |
| 33 | Yes | N/A | N/A |
| 34 | Yes | Longer waiting time | N/A |
| 35 | Yes | N/A | N/A |
| 36 | Yes | Longer - for me it is okay, even though it takes time it is good what they are doing. | And you find that now hospitals are clean. |
| 37 | Yes | N/A | They treat you quickly and wear mask. |
| 38 | Yes | N/A | Yes, They wear mask, protective clothes which they were not using before and the way they handle patients is totally different because they handle in shifts to prevent overcrowding  The doctors are really concerned unlike before corona pandemic. They test your condition then ask if maybe you have a family member who might have Covid-19 symptoms .They even provide masks at the hospital. |
| 39 | Yes | N/A | The health providers had there mask and gloves on but they ensured they were keeping distance while addressing us. |
| 40 | Yes | N/A - unclear | When consulting the doctor, the door is closed and you talk, only difference is the distance but it is just ok for me. |
| 41 | Yes | Short-You will not stay in the hospital for long because they also need space. | That wearing masks even nowadays they do not handle you the way they used to do, sanitizing nowadays it is a must to sanitize b they do handle you like before, they are so different.  th |
| 42 | Yes | Short - Not really I never waited for long to be served | doctors were in protective clothes and mask. |
| 43 | Yes | Same | They attended to me quickly just as it used to be before. |
| 44 | Yes | N/A | Health workers are more vigilant when attending to the patients unlike before when they  weren’t that much cautious such as observing good hygiene all the time.  -social distancing has enabled patients to be more free to the doctors and tell them  what they are really undergoing through because in the past, patients could be congested in  one room and make the other patient fear to speak up what he or she is going through. |
| 45 | Yes | Same waiting time | -every health  worker is observing all the hygienic measures put in place thus the patients feel safer. |
| 46 | Yes | Same waiting time | -workers are more cautious |
| 47 | Yes | Same waiting time | N/A |
| 48 | Yes | N/A | N/A |
| 49 | Yes | Same waiting time | N/A |
| 50 | Yes | Same waiting time | N/A |
| 51 | Yes | Longer waiting time. | N/A |
| 52 | Yes | Short waiting time | N/A |
| 53 | Yes | Short - No because they were reducing the number of people | N/A |
| 54 | Yes - they are only taking a few people. | Long - You will wait because you see the way we are being served one by one is how you protect yourself. | N/A |
| 55 | Yes - Only one person was going in at a time. | Same waiting time | N/A. |
| 56 | Yes - except for the protective measures put in place. | Short waiting time | N/A |
| 57 | Yes - restriction in the delivery rooms.  Now, it is different from before. Because now even if let's say you are going to deliver, you go alone or if you're going with someone, they are taking you and coming back. They leave you by yourself even if you have delivered. Mostly they restrict people from coming in even in that room that you are in. | Long waiting time | N/A |
| 58 | Yes | Short waiting time | N/A |
| 59 | Yes - What I noticed is that people are not crowded as before. | N/A | N/A |
| 60 | Yes | Short waiting time | During this time of corona virus the services are very fast and nurses are at your services people sit on one bench two mothers taking weight of the baby is not allowed because of congestion of mothers in one room. |
| 61 | Yes | Shorter waiting time | N/A |
| 62 | Yes | N/A | N/A |
| 63 | Yes | Shorter waiting time  the process has changed, it’s not like before where a person could spend more time waiting to be served. | N/A |
| 64 | Yes | Shorter waiting time | The nurses are not many like it used to be they use to spend time when they were together while patients waited to be served. |
| 65 | Yes | Shorter waiting time  I observed is the service is very fast there is no waiting for a long time sometimes you can find you’re the only person in the facility , | N/A |
| 66 | Yes | Same waiting time | N/A |
| 67 | Yes | Longer waiting time | N/A |
| 68 | Yes | Longer waiting time | N/A |
| 69 | Yes | Longer waiting time | N/A |
| 70 | Yes | Shorter waiting time | N/A |
| 71 | Yes | Shorter waiting time | N/A |
| FREQUENCY | YES- 71 **(100%)** | SHORTER- 23 **(32%)**  LONGER- 20 **(28%)**  SAME- 14 **(20%)**  N/A- 14 **(20%)** |  |

**THEME 4: EFFECTS/ CHALLENGES OF LOCKDOWN AND CURFEW**

| **Respondent ID** | **Effects on Health** | **Effects on relationship with partner** | **Effects economically, financially, business etc** | **Other Challenges** | **Positive Effects** |
| --- | --- | --- | --- | --- | --- |
| 1 | Good health | No disagreements | Lost job | The police are arresting the citizens aimlessly just because some of them don’t have masks not knowing that some of them can’t afford to buy a mask. | N/A |
| 2 | N/A | No disagreements | -No money for food  -No money for rent | This lockdown has made it so that you can't visit each other. | N/A |
| 3 | N/A | Disagreements and fighting with husband | -Work has been affected due to curfew hours (has an mpesa shop) | -Yes because you know someone comes and quarrels with you and maybe they beat you and they know there is nowhere you will go because there is lockdown. And if it wasn't there if you say go once they leave you go. But now there is lockdown, where will you go? You just have to persevere and stay there | N/A |
| 4 | Good health | Disagreements with partner- “That quarreling in the house. You stay you quarrel and sometimes you find you are fighting even if you don't want to.” | -Had to close her job (selling clothes)  -food is another one you find sometimes it's there sometimes it's not there.    -has not paid rent in 2 months | Food also I have to go for it early. I get into the house early. I stay in the house. Those other times you see I used to leave and come in even at like 8… 9 there. | N/A |
| 5 | Good health | N/A | -Closed down business | -Has developed stress because she has no money and has 4 children to take care of. | N/A |
| 6 | Good health | No disagreements | -prices of goods has gone up  -Sometimes we don’t ok consume the way we used  to, we have to minimize on the expenditure on the house, food, whatever, yeah.  -low income on husband’s side  -challenges paying rent | N/A | N/A |
| 7 | Good health | No disagreements | -challenge buying food | N/A | For me this lockdown and curfew has made me spend more time with my  partner because in the past he used to come back home late. |
| 8 | Good health | No disagreements |  | N/A | N/A |
| 9 | Good health | No disagreements | -inadequate funds to pay rent  and cater for the basic needs | N/A |  |
| 10 | Good health | No disagreements | Forced to close down business because there are no customers | N/A | N/A |
| 11 | Good health | No disagreements | Closing businesses | N/A | N/A |
| 12 | Good health | No disagreements | Lack of food and money to pay rent | N/A | N/A |
| 13 | Good health | Disagreements leading to divorce | -Inadequate food  -no jobs | N/A | N/A |
| 14 | Good health | N/A | -people are unemployed hence no money to acquire food | Less working hours due to curfew | N/A |
| 15 | N/A | N/A | -husband has been retrenched and salary reduced  -can longer save money  -essential items are expensive  -no jobs | The government is not helping its people  - if a baby gets sick at night, with the curfew on it is very difficult to access  healthcare | N/A |
| 16 | Bad health- mild depression | N/A | -Getting food and other daily necessities is very difficult.  -husband laid off from work  -frustration mainly due to  restrictions preventing me from attending my daily work; | N/A | N/A |
| 17 | N/A | N/A | N/A | N/A | N/A |
| 18 | Good health | No disagreements | -my husband who has a cybercafé and the customers  have reduced because of the lockdown and also the curfew time hours where he is forced to  close the business earlier than before. | N/A | N/A |
| 19 | Good health | No disagreements | The businesses aren’t that profitable due to decreased working hours  -Inadequte food  -unemployment | N/A | N/A |
| 20 | Good health | N/A | N/A | N/A | N/A |
| 21 | Good health | N/A | -challenge acquiring food  -unemployment | N/A | N/A |
| 22 | Good health | N/A | -no food, forced to sleep hungry at times  -problem going to the market to get things to sell | -forced to close work early due to curfew  - | N/A |
| 23 | Good health | Disagreements | -Unemployment  -Problem getting food at times  -goods are expensive | -lock down has affected her movement, she would have traveled to the countryside | But on the  other hand that curfew there was a way it was helping, according to the way I have heard  families talking, it has helped families...curfew it made it  mandatory for someone to be in the house |
| 24 | Good health | N/A | -no employment- used to do laundry but now clients do not allow her into their homes. Son cannot also go t work  -no money to pay rent  -problem feeding children | N/A | N/A |
| 25 | N/A | N/A | Unemployment | -can't go visit children who are in the countryside due to lock down | N/A |
| 26 | N/A | No disagreements | -time of work is limited  -there is no money eating  -paying house rent is a problem | N/A | N/A |
| 27 | Good health | Disagreements  -It is brought about by the lack of money, and the fact that there’s no  job. The children also want to eat and there is no money, they come to me and  when I go to him and he doesn’t have it becomes a problem. | -My husband works in construction and at times he has been called for  jobs outside of Nairobi and as a result of the lock down he can’t go. This has affected us financially | Experiences depression at times | N/A |
| 28 | Bad health- has been experiencing depression | N/A | -Nobody wants to employ  someone at this time even to do casual work such as do their laundry.  -there is shortage of  food and I have a small baby. | N/A | N/A |
| 29 | Good health | N/A | -unemployment  -shortage of food items | N/A | N/A |
| 30 | Bad health | N/A | -shortage of food | N/A | N/A |
| 31 | N/A | N/A | -No finances  -My husband is in transport business so at times he finds job at 9pm or 11pm but he can’t  go because of the curfew. | N/A | N/A |
| 32 | Good health | N/A | -Facing economic problems | I just wanted to travel to the rural home but  because of the lockdown I can’t travel. | N/A |
| 33 | Good health | N/A | With me things have really changed because no more going to work, so even  getting meals is difficult. | Experiencing some stress | N/A |
| 34 | Good health | No disagreements | my husband, the casual job they used to do they were  stopped | N/A | N/A |
| 35 | Good health | N/A | The only problem is that my husband was locked down  in Nairobi and the baby has not seen him for two months. | N/A | N/A |
| 36 | Good health | No disagreement-happy to spend more time with the husband. | Short business time- At first it was affecting people’s businesses because they had to close early but now at least the curfew was extended to 9pm. | N/A | And then the lockdown I think it is good because if it was not there I feel this disease would have spread everywhere. |
| 37 | N/A | No disagreements | - many people don't go to work, -we can even sleep hungry. | N/A | N/A |
| 38 | Good health - With me I am just okay with no stress | N/A | N/A | Because of the lockdown I sometimes lack proper communication with my husband because it forces them to stay more days at work before he comes back home. | N/A |
| 39 | Good health | N/A | N/A | Stress due to fear of contracting covid-19 | N/A |
| 40 | Good health | No disagreements | Short selling times - supplies go bad | Police harassment - Where I stay, some of the people were not obeying the curfew. As a result, the police would come and discharge teargas which would come into the house and being that I underwent CS I couldn’t sneeze. So, my sister would hold my nose as I hold my baby’s nose. That is the biggest challenge that I have experienced. | N/A |
| 41 | Good health | N/A | Reduced working times - us we are hustlers so you find time is over you have not finished the work you had may the customer who was coming has delayed and has not given you money so you stay like that. | N/A | N/A |
| 42 | Good health | N/A | N/A | N/A | N/A |
| 43 | Bad health - depression | No disagreements | -Unemployed  -unable to afford medication for diabetes  -increased debts | -loss of businesses  -joblessness  -lack of income | N/A |
| 44 | Good health | Disagreements  -we are disagreeing on many things during this COVID -19  pandemic since each one of us has lost their jobs therefore everyone is struggling to put  something on the table and this makes us disagree most of the time. | -her and husband lost their jobs  -worried about feeding her family | -has been experiencing stress  -restriction on movement, she cannot go out of town to find cheaper food for her family | N/A |
| 45 | Good health | No disagreements | -lost job  -challenge putting food on the table | N/A | N/A |
| 46 | Bad health | No disagreements | -loss of jobs | -I’m stressed with what my children will eat and yet  I don’t have a job so far | N/A |
| 47 | Good health | No disagreements | -businesses have really gone down in terms of operation even others have  been forced to close up  -prices of goods especially food stuff has gone up | N/A | N/A |
| 48 | N/A | N/A | -husband lost job | -has been experiencing stress | N/A |
| 49 | Good health | N/A | -business has gone down  -challenge getting food | -stuck in the countryside with children and husband is in Nairobi | N/A |
| 50 | Good health | No disagreements | -no money  -challenge getting food  -challenge getting a job | -movement restrictions | N/A |
| 51 | Bad health | disagreements | -financial problems | -affected psychologically by disease because the people you could talk to when you have problems you can’t  see them. You are just alone. Calling them is not enough. | N/A |
| 52 | Good health | No disagreements | Financial constraints- unable to provide even pampers for the baby | N/A | N/A |
| 53 | Good health | No disagreements | -no jobs | -That putting on masks all the time is boring. Someone can’t breathe well, they  can’t speak in peace… | N/A |
| 54 | Good health | No disagreements | -businesses has been affected with the curfew | -restricted movement due to curfew and lock down | N/A |
| 55 | Good health  -but has lost weight | No disagreements | Husband lost his job  -challenge getting food  -prices of goods, especially food has gone up. | N/A | N/A |
| 56 | Good health | No disagreements | -challenge paying rent | N/A | N/A |
| 57 | Good health | No disagreements | -has affected husband’s business-has a hotel | -children stuck in the countryside due to the lock down | N/A |
| 58 | Good health | N/A | -financial challenges | N/A | N/A |
| 59 | Good health | Disagreements  -misunderstanding is there because my husband is in the house throughout he  does not go to work. | -challenge getting food | Restricted movement  -essential commodities prices are hiked transport we pay double. This time Covid have really changed the way people are leaving there is no money. | N/A |
| 60 | N/A | NA/ | -financial constraints | -restriction of movement  -cannot visit friends and family | N/A |
| 61 | N/A | N/A | -no money  -sometimes can’t afford food  -can't pay rent  -husband locked out of town | N/A | N/A |
| 62 | N/A | Disagreements | -no jobs | -am stressed in that sometimes my baby don’t get enough milk to breast feed, all this is happening because of lockdown and curfew.  -if you don’t put on mask you’re arrested on the spot and you pay some money | N/A |
| 63 | N/A | N/A | -husband cannot go to work  -they sleep hungry sometimes  -I used to wash for people cloths but now I can’t go because of corona | people don’t go to church and children don’t go to school | N/A |
| 64 | N/A | -no disagreements | N/A | -I would wish to travel and I can’t because of the lockdown  -children are not going to school | N/A |
| 65 | N/A | N/A | N/A | The bad thing is that the policemen throw tear gas to people in the community children suffocate | N/A |
| 66 | Good health | N/A | -lost her job  -getting something to eat is a challenge  -money to pay rent is a challenge | N/A | N/A |
| 67 | Good health | disagreements | -financial constraints  -cannot go to work  -husband no longer goes to work  -challenge with rent  -challenge with food | -It affected me, because I was to go back to my rural home but I could not go. | N/A |
| 68 | Good health | No disagreements | - husband lost his job  -challenge feeding children | -can no longer visit relatives | N/A |
| 69 | Bad health  -You can persevere its not like before someone used to get some slight discomfort you run to the hospital at least now someone just perseveres to see if there is a way you can recover. | N/A | -challenge getting food | -husband locked out of town, has challenges taking care of new born baby alone | N/A |
| 70 | Good health | Disagreements | -challenge getting food  -challenge paying rent  -husband was laid off | N/A | N/A |
| 71 | Good health | No disagreements | -husband lost his job  -challenges getting food | N/A | N/A |
| FREQUENCY | GOOD HEALTH- 49**(69%)**  BAD HEALTH- 7 **(10%)**  N/A- 15 **(21%)** | DISAGREEMENTS- 11 **(16%)**  NO DISAGREEMENTS- 30 **(42%)**  N/A- 30 **(42%)** |  |  |  |

**THEME 5: GOVERNMENT AND COMMUNITY SUPPORT**

| **Respondent ID** | **Practices by community to prevent spread of COVID** | **Support from community to prevent spread of COVID** | **Support from government to prevent spread of COVID** | **Leadership- yes/ no** |
| --- | --- | --- | --- | --- |
| 1 | Yes  -social distancing  -wearing masks  -hand washing | N/A | Yes  -given soap and water once | No |
| 2 | No.  Even as we speak where I am right now there is none because there's a field nearby where people come to drink and get drunk. So I can't say there is. There is none. | No | No | No |
| 3 | No | No | No | No |
| 4 | Yes  -washing hands before entering gate | No | No | No |
| 5 | Yes  -sanitizing  -washing hands  -keeping distance | Yes- The community we are just helping each other by washing hands and wearing  masks, if I have something I give my colleague and if I don’t have they give me. | No- The government said they are giving but us its like they have not  gotten to us. | No |
| 6 | No  - Like right now in Kayole they are not taking the measures that seriously. Because  when you go around there are just children playing around,people interacting they are not  keeping that distance, social distancing and masks some of them are not putting them on. | No  - I don’t see even at shops there’s no water  to clean yourself so it’s like they are not taking it that serious. Before it was serious but right now  people are just reluctant. | Yes-  But I think once, I heard people  out there saying that the government, people came with sanitizers saying that they were from  the Government. | No |
| 7 | Yes  -frequent washing of hands  -wearing masks | No | No | No |
| 8 | Yes | No | No | No |
| 9 | Yes | Yes | Yes- most neighbors have received funds from government but not her | Yes  -Chairlady  -She has created some employment opportunities so that those who have lost  their jobs because of coronavirus can get something to keep them occupied |
| 10 | Yes  -frequent washing of hands  -wearing masks  - | Yes | No | Yes  -Area Chief  He makes patrols daily to make sure that the residents in his location are wearing  masks and they aren’t having unnecessary gatherings. |
| 11 | Yes | No | No | Yes  -Husband  -He has both face masks to every person in the family and he keeps on telling the  neighbors to be cautious and keep on wearing face masks and frequently washing their hands  because this disease is real. |
| 12 | Yes | No | No | No |
| 13 | Yes | No | Yes- provided water | No |
| 14 | Yes | No | No | No |
| 15 | No  Nothing is done in our community we put measures on our own | No | No | No |
| 16 | Yes  -They are also vigilant that no visitor is permitted in the Estate.  -washing hands  -wearing masks | Yes  -the community are making sure that people adhere  to the laid down procedures of wearing mask, washing hands and social distance. The  community also has remained vigilant and has placed restrictions on visitors. | No | Yes  -Woman herself  -I have been doing this. Where I was working they had trained me in handling  such cases. |
| 17 | Yes | No | No | No |
| 18 | Yes | N/A | Yes  -I heard from my neighbors that they received food from the government but I  haven’t seen it on my side. | No |
| 19 | Yes | No | No | No |
| 20 | Yes | No | No | Yes  -Elder sister  -She keeps on reminding the community members to observe the safety  measures and also telling them not to look down upon COVID-19 because the disease is real  and it is killing people. |
| 21 | Yes | No | Yes  I haven’t seen the support on my side but I usually hear that there is  distribution on masks and food which I haven’t received so far. | No |
| 22 | Yes | No | No | Yes  -Chief  -Sensitizing people about disease |
| 23 | Yes | No | No | No |
| 24 | Yes | Yes  -I have received two packets of maize flour and one litre of cooking oil from the  church, | No | No |
| 25 | Yes | Yes  -one day we were given a bar of soap with an organization called SHOFCO. | No | No |
| 26 | Yes | Yes  what I know every  business in the community have put a place where there customers wash they hands before and after  purchasing. | No | No |
| 27 | Yes  -washing hands  -wearing masks | No | No  I have never seen or received any support from either the community  or the government. One time they wrote our names down but nothing happened  after that. | No |
| 28 | Yes | No | No  We registered for government assistance, but nothing has come through. | Yes  -The Area Chief.  -He ensures that there is law and order and that the  community takes precaution by washing hands and the use of face mask in crowded  places. |
| 29 | Yes | Yes | Yes  All communal areas are cleaned regularly with the use of fumigation and  sanitizing fluids. | No |
| 30 | Yes | Yes | No | Yes  -Area Chief  -He ensures that there is law and order and that the  community takes precaution by washing hands and the use of face mask in crowded  places. He is always seen patrolling the area. Visitors from outside the area are not  allowed to come into the community. |
| 31 | Yes | No | No | No |
| 32 | Yes | Yes  There was a list that we filled at Ruben hospital to be given three thousand .That  is the only support we have received so far.. | No | No |
| 33 | Yes | Yes  For me I have received a little support from “Give direct” funds and am so  thankful for that. | No | No |
| 34 | Yes | No | No | No |
| 35 | Yes | No | No | Yes  Area Chief  He ensures children are wearing  masks as they go to market places. |
| 36 | Yes | No | Yes - one time they were spraying shops to sanitize them | No |
| 37 | Yes - water and sanitizer | No | No | No |
| 38 | Yes - hand washing, avoiding unnecessary movements | No | No | No |
| 39 | No- In our area people are ignoring the measures put in place by the Government like putting on masks. | Yes | No | No |
| 40 | Yes - water everywhere for washing hands. | No | No | No |
| 41 | Yes | No | Yes - water tanks and masks | No |
| 42 | Yes | No | No | No |
| 43 | Yes - frequent washing of their hands, wearing masks, sanitizing and frequent cleaning of their surfaces where they are living. | No | Yes - food | Yes - youth are cleaning community surfaces and enforcing good hygiene |
| 44 | Yes | Yes | Yes | Yes  MCA  -has coordinated  the construction of a tent with the area where each one of us can go and wash their hands  freely. |
| 45 | Yes  -Only a few people are observing the  preventive measures put in place by the government such as wearing masks and washing our  hands frequently; most of the people aren’t complying with rules put in place. | No | No | No |
| 46 | Yes | No | No | No |
| 47 | Yes | Yes  -community members are educating each other so that they can have full  knowledge about the disease. | Yes  -The government is distributing face masks and sanitizers to the people and also  facilitating the educating of Dandora residents to know all the facts about COVID-19 in order to  reduce its spread in the community. | No |
| 48 | Yes | No | No | No |
| 49 | Yes | No | No | Yes  MCA  -giving people masks and sanitizer |
| 50 | Yes | Yes  -given financial aid | No | No |
| 51 | Yes  Here at my community people are not very serious but at least I can see they are  washing their hands. But things like distance and staying in the house very few have  maintained. | No | No | Yes  -”nyumba kumi”  -has tried a little to get people inside during the curfew. |
| 52 | Yes | Yes  -the young boys they are doing cleaning.  -There’s just this organization that’s calling itself sofco.  They are the ones we have seen removing soap, and taking it round the houses, door to door. | No | No |
| 53 | Yes | No | No | No |
| 54 | Yes | No | No | No |
| 55 | Yes | No | No | No |
| 56 | Yes | No | No | No |
| 57 | Yes | No | No | No |
| 58 | Yes  -Tanks are put everywhere people wash their hand before entering their houses. | No | No | No |
| 59 | Yes | Yes  What I know is that people have put up points for washing hands, people are getting food  and some don’t receive | No | No |
| 60 | Yes | Yes  organizations come to our community to give advice  on how people should live with coronavirus. | No | Yes  -Our caretaker is the one who educate us on what we are supposed to do for example if  you climb the staircase we should not support ourselves and to wash our hands |
| 61 | Yes | No | No | No |
| 62 | Yes | No | Yes  I hear from people food is distributed at night to those who are known to the people concerned. | No |
| 63 | Yes | Yes  Ruben Centre distributes food | No | Yes  Area Chief  -he is trying we get clean water, soap and mask distributed by SHOFCO, |
| 64 | Yes | No | No | No |
| 65 | Yes | Yes | No | No |
| 66 | Yes | Yes  -received Flour, rice, sugar, cooking oil, bread too. | Yes | Yes  Chief  -has tried to restrict the entry of visitors into the community  MCA  -He used to call and warn people about letting strangers in and encouraging people to report anyone who does it.  Landlords  -They were putting notices on their plots warning outsiders not to enter. If you are found to have allowed people to enter you are taken to quarantine. |
| 67 | Yes  -washing hands | No | No  I have only seen people receiving things on tv but I have never heard or seen anyone from here receiving any government relief. | No |
| 68 | Yes | Yes | Yes  I was supported with two loaves of bread, I was supported with bathing soap, and one packet of baking flour and one packet of maize flour. | No |
| 69 | Yes | No | No | No |
| 70 | Yes | Yes | Yes | No |
| 71 | Yes | No | Yes  They were assisted I saw money being sent from the government | No |
| FREQUENCY | YES- 66 **(93%)**  NO- 5 **(7%)** | YES- 23 **(32%)**  NO- 46 **(65%)**  N/A- 2 **(3%)** | YES- 15 **(21%)**  NO- 56 **(79%)** | YES- 16 **(23%)**  NO- 55 **(77%)** |

**THEME 6: RECOMMENDATIONS TO GOVERNMENT**

| Respondent ID | Recommendation |
| --- | --- |
| 1 | - distribute masks and food because most people are jobless |
| 2 | -The first thing I'd like the gvt to do is put security. And after putting security these policemen should not accept bribes. Because you can find them collecting bribes and people continue to sit and drink without social distance.  - They should talk to the landlords to just reduce something even if it's 25% because it's not all landlords that reduced the rent |
| 3 | -If it's about restrictions they just put them the ones for protection but people just work so that they are not affected.  -Remove the lockdown |
| 4 | -Remove the lock down so she can travel to her rural home  -help with finances for household needs |
| 5 | -government should give grants  -They should increase the number of hospitals and add beds at the hospitals and enough equipment so that at least when someone is taken there you will be attended to well.  -give food to those that are unable |
| 6 | -first of all they would have done this testing door to door like the way they did census and separated those who have the disease from those who do not  -have more isolation centres  -provide essential items to the community and also speak to the landlords about the rent issue |
| 7 | -The government should try and provide masks and sanitizers because not all people can afford to buy them.  -the government should try as it can to help us in paying rent  -distribute food to the people |
| 8 | -the government needs to distribute funds to the unemployed residents  so that they can start small businesses and be independent to themselves.  -provide water and food to residents |
| 9 | -the government needs to continue distributing funds so that the citizens  can be able to buy masks so that they can protect themselves from getting infected  -reduce taxes and the price of essential goods. |
| 10 | -the government needs to distribute funds to the unemployed residents so  that they can start small businesses and be independent to themselves. |
| 11 | -the government should just open up so that we can have our businesses  -provide food and water to community |
| 12 | -Distribute masks among the residents  -try and reduce the prices of goods sold in the market and also the taxes imposed to the citizens. |
| 13 | -The government should just open up the country and remove the curfew so that the citizens can acquire jobs so that we can be independent.  -it should look into the pricing of products and reduce the prices so as to reduce the expenses. |
| 14 | -I would like to request the government to distribute masks, sanitizers and water.  -I would also like the government to distribute food to the residents of Dandora because many are dying of hunger  -remove the curfew so that people can freely work at any time of the day. |
| 15 | -The government can’t control this disease it upon us to put on mask because some people when they see the policemen it’s when they wear mask and they don’t know if you don’t wear mask you put your family at risk (Personal Responsibility) |
| 16 | -My appeal to the government is to reach out to those in our community who  are not able to buy mask, sanitizer liquid, food and medicines. |
| 17 | -The government should continue with the lock down and curfew to reduce  new infection cases |
| 18 | -I think the government should just open up the country when the coronavirus  cases start to decrease. |
| 19 | -help citizens acquire their jobs so that they can be independent.  -do mass testing |
| 20 | -help those in need by supporting them to acquire food. |
| 21 | -to provide food for the residents of Dandora  - look for a way in which they can reduce the cost of rent.  -find a vaccine so that life can go back to normal |
| 22 | -government to just give her something |
| 23 | -reduce the price of goods to make it affordable to the people other than providing grants/ food which does not get to the people |
| 24 | -to be given masks and sanitizers for protection  -to be assisted with food  - helped to pay rent |
| 25 | -give people food  -ease restrictions so people can move |
| 26 | -The government should give people masks  -open the roads for people to travel .  -everyone to take care of themselves. |
| 27 | -They should have locked down Nairobi earlier to help reduce the  spread of the virus. |
| 28 | -I would urge the government to find ways to allow workers to go back to  work, but maintain the preventive directives |
| 29 | -I would urge the government to find ways to allow workers to go back to  work |
| 30 | -I would urge the government to find ways to employ more doctors who  can treat this disease.  -The Government should do more testing so that those who are infected can be treated. |
| 31 | -They should help so we don’t commute and stay at one place. |
| 32 | -provide for us some food stuffs  -talk to the landlords to reduce the rent since most of us have no jobs. |
| 33 | -The government should continue with the lock down and curfew to reduce new infection cases because if the lockdown is removed some people may travel to the rural areas which will increase the number of infection cases  -The Government should see the issue of food because not all are at work during this pandemic. |
| 34 | -We can continue with the lockdown and the curfew, but they open the jobs. |
| 35 | -If the government could find a vaccine so that life can continue. |
| 36 | -give aid to those people who are unable to help themselves completely, those ones who have lost their jobs.  -They should also provide water and soap. Some people do not have that and life is hard.  -And then they should not let people travel everywhere. They should not let people travel until this disease has reduced. |
| 37 | -The government should give us food,  - lower the economy to us because everything is expensive. |
| 38 | -The government should continue with the lock down and curfew to reduce new infection cases but at least try to find medication for the virus for things to get back normal because we are suffering due to lack of food and money. |
| 39 | -: I would like the government to continue with measures but at least open up the churches. |
| 40 | -encourage people to continue wearing masks and maintain washing their hands. |
| 41 | -It should give masks the way they are saying they will give out masks in schools and those things of washing hands just that.  - help the poor if someone gets infected they treat them |
| 42 | -The government should open up the lockdown within Nairobi but maintain the lockdown around its border |
| 43 | -: I would like the government to provide food for those who aren’t able to put something on the table.  - the government should coordinate with the Ministry of Health and try to get the vaccine of this dangerous disease. |
| 44 | -The government should try and distribute food to those who are less fortunate because they aren’t able to provide food for themselves because they are unemployed. |
| 45 | The government should continue enforcing the rules put in place by the MoH such that every resident here at Lakisama should wear masks and observe social distancing when interacting with other people.  -I would like the government to do for us is to try and distribute food amongst the people  -also help in the covering of rent expenses. |
| 46 | -The government should distribute some funds to us so that we can be able to cater for our daily expenses, especially food and rent.  -I think the government should work hand in hand with the Ministry of Health so that they can be able to get the vaccine for coronavirus. |
| 47 | -The government needs to distribute to the less fortunate and the unemployed.  -also it should ensure that the people are fully trained and have full knowledge about the  disease in order to reduce its spread. |
| 48 | -They should first help people to feed their families  they should not lift the lockdown because the disease will spread quickly because people will be congested. |
| 49 | -I would have liked the government to give support in aid  -That lockdown should still continue because when we say now this lockdown should be removed, people will escape to the village and they  will spread that disease. |
| 50 | -lift the lockdown  -provide people with masks and sanitizer  -provide food  -ensure children are able to learn online |
| 51 | -educate citizens about corona  -give financial aid |
| 52 | -They at least they give us even if it's any project that can uplift us. Us like women who have children you know not all can do that cleaning job those who have time so women who have children should really be looked after.  -Health wise it's just that soap mostly and water. You water also has an issue because you buy at 5ahilling or ten shillings and if you don't have that income it becomes very hard to get that water. So now we were seeing at least if they can help us even if it is water at least we can try and help ourselves with hygiene. |
| 53 | -if they can help us even if it is water at least we can try and help ourselves with hygiene.  -come up with projects that people can participate in and make some money |
| 54 | -allow people to go back to work |
| 55 | -They just release money.  - They just bring a cure.  -Food...you know sometimes they are dying of hunger. |
| 56 | -They would just be providing us with food because I saw like they had given but it's like not everyone was given… |
| 57 | -talk to these landlords, they reduced the rent for us like even half  -he would have just removed the lockdown  -encourage people to practice the protective measures |
| 58 | -they should stretch their hand to everyone in the community not only to people with disability even us we don’t have they should look at each corner. because it’s not everyone who is getting food, |
| 59 | -would the government to do out with lockdown you can go to other counties to look for employment, |
| 60 | -The government should give its people soap sanitizer and food, most of the items we use daily is very expensive. |
| 61 | -The government should open churches for people to worship  -if you have any problem you should travel to your rural home |
| 62 | -people who have low income to be given support |
| 63 | -the government should continue with the lockdown and curfew or else everybody can get coronavirus so they should give people food |
| 64 | -The lockdown should continue and leaders should go round sensitizing the community about corona virus |
| 65 | -The government should buy for people mask and sanitizer  - the government should talk to the owners of the house to make them understand what people are going through, there is no money for paying for house rent |
| 66 | - I think the best way to help curb this disease is by extending the lockdown and the curfew. |
| 67 | -The government should have helped people with some cash, even if it's just Ksh. 500, it would have really gone a long way. |
| 68 | -the government to open up churches...you know we are supposed to just pray so that that disease does not get to us.  -they open up jobs so people go to work.  -reduce prices of goods. |
| 69 | -there should have been this lockdown completely until this disease is able to be eradicated or reduced.  -if they help us with something like food at least someone can relax and stop worrying. |
| 70 | -they should go door to door and test people.  -send money to people |
| 71 | -financial assistance |
|  | Summary Food = 21/71 = 30%, financial assistance = 10/71= 14%, Rent = 5 , (7%) reduction in price of goods = 5( 7%) and jobs = 6 ( 8%) |
